# Disorder at the Start: The Contribution of Dysregulated Translation Initiation to Cancer Therapy Resistance

**DOI:** 10.3389/froh.2021.765931

**Published:** 2021-12-07

**Authors:** Gulshan Sunavala-Dossabhoy

**Affiliations:** Department of Biochemistry and Molecular Biology, Louisiana State University Health and Feist Weiller Cancer Center, Shreveport, LA, United States

**Keywords:** eIF4E, 4EBP, eIF4A, eIF2A, eIF2, cap-dependent, translation, TLK1

## Abstract

Translation of cellular RNA to protein is an energy-intensive process through which synthesized proteins dictate cellular processes and function. Translation is regulated in response to extracellular effectors and availability of amino acids intracellularly. Most eukaryotic mRNA rely on the methyl 7-guanosine (m7G) nucleotide cap to recruit the translation machinery, and the uncoupling of translational control that occurs in tumorigenesis plays a significant role in cancer treatment response. This article provides an overview of the mammalian translation initiation process and the primary mechanisms by which it is regulated. An outline of how deregulation of initiation supports tumorigenesis and how initiation at a downstream open reading frame (ORF) of Tousled-like kinase 1 (TLK1) leads to treatment resistance is discussed.

## Introduction

**Figure 1:**
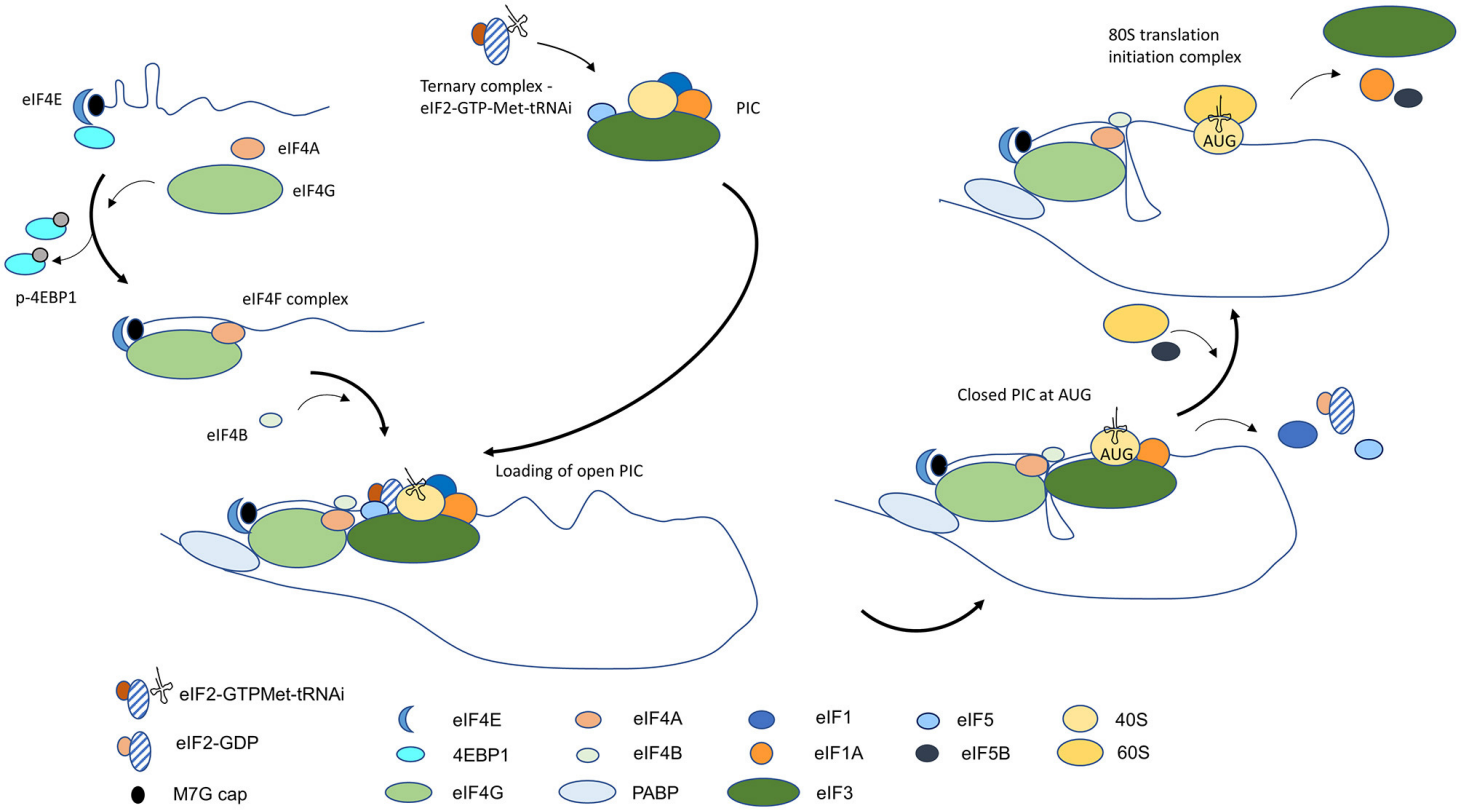
Diagrammatic overview of conventional cap-dependent translation initiation in eukaryotic cells. Translation initiation begins with the assembly of the ternary complex and PIC and binding of 5′ mRNA cap by the eIF4F complex. Unwinding of mRNA near the cap by eIF4F facilitates loading of the open state PIC that is conducive to scanning. A conformational change to a closed state occurs on recognition of the start AUG, and with the entry of 60S ribosomal subunit and the formation of the 80S translation initiation complex, protein synthesis begins.

The process of translation initiation begins with the formation of two protein complexes that occur in parallel and converge at the 5′ end of the mRNA. The ternary complex—which comprises of eukaryotic translation initiation factor 2 (eIF2), GTP, and initiator methionyl-tRNA (Met-tRNA_i_)—is the preliminary step in the assembly of the 43S pre-initiation complex (PIC). Together with the 40S ribosomal subunit and eukaryotic translation initiation factors, eIF1, 1A, 3, and 5, the ternary complex binds to form the PIC. Independently, assembly of a protein complex occurs at the 5′ end of the mRNA through recognition of the m7G cap by the cap-binding protein, eIF4E. The RNA helicase eIF4A and the scaffolding subunit eIF4G recruitment results in the formation of the eIF4F complex. The binding of eIF4B to eIF4A stimulates the unwinding of the mRNA immediately downstream of eIF4F, which facilitates the loading of the PIC [1]. Through interaction with eIF4E and the poly A binding protein (PABP), eIF4G bridges the 3′ and 5′ ends of the mRNA forming a closed-loop conformation that aids in spatially localizing the translation machinery for subsequent rounds of protein synthesis on the same translated mRNA [1] (Figure 1).

Recruitment of the PIC is facilitated by eIF4A-mediated unfolding of mRNA and the affinity of eIF3 for RNA and for eIF4G. The PIC is loaded on the mRNA in an open conformation, which is permissive to scanning, to locate the start AUG trinucleotide that can base-pair with the complementary sequence in Met-tRNA_i_ [2]. The search process occurs linearly in a 5′-3′ direction and is presumed to be due to eIF4A-facilitated unwinding of RNA structures at the leading edge of PIC and the presence of eIF4B at the trailing end to restrict movement of PIC in reverse. The identification of the first AUG in an optimal sequence context initiates stable pairing with the anti-codon in the Met-tRNAi and it leads to the eviction of eIF1 and the hydrolysis of eIF2-GTP to eIF2-GDP by eIF5. The full-engagement of the Met-tRNAi at the P site in the 40S ribosomal subunit results in a change in PIC to a close state which halts further scanning of the mRNA [2]. Subsequent release of eIF2-GDP and eIF5 makes way for the joining of the 60S ribosomal subunit, catalyzed by GTPase eIF5B, and for the formation of the 80S translation initiation complex. The dissociation of eIF5B-GDP and the departure of eIF1A signals entry of the translation initiation complex in the protein synthesis phase (Figure 1) [1–3].

Translation initiation is a highly regulated cellular activity that occurs in response to the availability of molecular factors, nutrients, and hormone and stress signaling. It is tightly controlled at multiple steps in the process and described here are the major regulatory nodes and our understanding by which deregulation of initiation shifts the cellular proteome to being conducive to tumorigenesis.

## Regulation of Ternary Complex Formation

The control of translation at the ternary complex assembly is at one of the earliest of stages of translation initiation. eIF2 is essential to the loading of Met-tRNAi onto the 40S ribosomal subunit for the assembly of the 43S PIC. Since Met-tRNAi has a higher affinity for GTP-bound eIF2 than GDP-bound, the GDP-GTP exchange factor, eIF2B, is an important player in regulating ternary complex formation. eIF2 is a heterotrimeric protein composed of α, β, and γ subunits. Cellular kinases activated by stress such as protein kinase R-like endoplasmic reticulum kinase (PERK) in unfolded protein response, GCN2 in amino acid deprivation, RNA-activated protein kinase (PKR) in viral infection, and HRI in metabolic stress can phosphorylate eIF2α at serine 51. The GDP-bound phospho-eIF2-Ser 51 avidly interacts with and sequesters eIF2B, reducing levels of unbound eIF2B for eIF2-GDP to eIF2-GTP recycling [4]. As a result, phosphorylated eIF2 tamps down global translation initiation in stress. Through interaction with eIF2 and protein phosphatase 1 (PP1), GADD34 facilitates the dephosphorylation of eIF2 by PP1 [5], which restarts protein synthesis in cell recovery.

Despite repression in global translation initiation in stress, protein synthesis from stress-response transcripts remains largely unaffected. There are two prevailing mechanisms by which translation of these transcripts occur. First, the incorporation of phosphorylated eIF2 in the PIC reduces scanning fidelity of the complex, leading to the bypass of the upstream start sites. ATF4 is a transcription factor critical to the expression of genes that drive a prosurvival cellular program in response to stress [6]. With 2 inhibitory upstream ORFs, translation of ATF4 is limited in non-stressed cells. However, phospho-eIF2 can contribute to leaky scanning, and initiation at downstream ORFs leads to protein synthesis such as of ATF4 that would otherwise not occur in homeostasis [7]. Additionally, sequestration of eIF2B by phospho-eIF2 leads to limited Met-tRNA-engaged PIC and the overriding of initiation at upstream ORFs. Through association of the scanning ribosomal 40S subunit with the ternary complex downstream initiates translation at the next ORF.

Second, the alternate initiation factor eIF2A competes with eIF2 for loading of initiator tRNA (tRNAi) on the 40S complex. eIF2 is the predominant player in translation initiation, but following its phosphorylation and sequestration, contribution of eIF2A to ternary complex formation increases significantly as it is refractory to eIF2 inhibitory kinases [8, 9]. Although a direct demonstration of interaction is lacking, eIF2A can recruit an alternative initiator tRNA, Leu-tRNAi, and initiate translation at non-AUG triplets such as CUG and UUG that would otherwise be discriminated against. As alternative ORFs do not contribute significantly to the translational landscape in non-stressed cells, an incremental increase in eIF2A can lead to a relatively large increase in alternate ORF expression.

Certain stress response transcripts evade translational repression through initiation at IRES or at sequence-specific elements in the 5′ UTR. Notably, a switch from cap-dependent to cap-independent translation of prosurvival factors, HIF-1α, VEGF, and BCL2, is induced by hypoxia [10]. Translation initiation occurs independent of canonical ternary complex and relies on eIF5B to deliver tRNAi.

## Length and Complexity of 5′UTR

When present in a favorable sequence context, translation begins at the first AUG near the 5′ end of the mRNA. However, length and structural complexity of the 5′UTR can influence the rate of translation initiation. An inverse relationship exists between translation initiation and length of the 5′UTR and its ability to self-anneal and form complex structures [3]. Self-assembly of single stranded RNA into stable secondary structures impedes movement of the PIC and limits initiation at the projected start site. Helicase activity of eIF4A is vital to deconvoluting short double-stranded mRNA at 5′ end for loading of the 43S PIC, and structured mRNA 5′ ends are reliant on eIF4A for efficient initiation. However, eIF4A is insufficient to unwind structures of high complexity during scanning and participation of cellular helicases such as DHX29 and DDX3 is vital to resolving the structures [11].

## Role of Sequence in Start Codon Selection

Traveling downstream from the 5′ end of the mRNA, the PIC scans the sequence base-by-base to locate the start site. Translation often starts at the first AUG that is encountered; however, its selection as a start site is dictated by the context in which the trinucleotides reside [12]. When present within the Kozak consensus sequence, G/ACCAUGG, the trinucleotides are considered to be in an optimal context for translation initiation [13]. In particular, the nucleotide at −3 position in relation to the AUG determines the efficiency of start codon selection and there is a preferential occupancy of an A in yeast and mammals [14]. Often AUG in a poor context escapes detection by the scanning machinery for another downstream that is in a better contextual reference frame. Non-optimal sequence context or near-similar start triplets such as CUG or UUG in a favored consensus can lead to inconsistencies in the use of the start codon and misguided translation initiation accounts for the diversity of protein isoforms. By computational analysis nearly half of the transcripts in mammalian cells have an upstream ORF, and ribosomal profiling suggests that many are translated in homeostasis [15]. In instances where downstream AUG(s) is in-frame without an intervening stop codon, leaky scanning at upstream AUG can result in protein isoforms that differ in the N-terminus region but are otherwise identical. N-terminal differing isoforms when sorted in separate cellular compartments can have distinct cellular targets and function. On the other hand, an upstream AUG residing in a near-optimal sequence context significantly limits initiation of the main ORF from the downstream site. Control of initiation through uORFs regulates the translation of tumorigenic proteins such ATF4 in homeostasis. In the event of a stop codon between the upstream and downstream AUGs or an intervening RNA sequence with a propensity to self-anneal into stable structures stalls PIC that skipped the upstream AUG, or ribosomes, attenuating the re-initiation at the downstream ORF and a paucity of the main protein isoform. Often, this is not the case in normal cells as ternary complexes are abundant and they reassociate to form a translation competent complex for initiation at the downstream start codon.

## Influence of Initiation Factors in Start Site Selection

Translation initiation factors that form the PIC also play important roles in the selection of the start codon [12]. Structural studies indicate that eIF1 interaction with Met-tRNAi allosterically prevents its precise engagement at the P site in the 40S ribosomal subunit and thus keeps the PIC in an open conformation that is conducive to scanning [16]. Imperfect fit of the Met-tRNAi is suggested to also discriminate against sub-optimal anti-codon pairing. With accurate base-pairing at AUG in preferred consensus, the release of eIF1 occurs and the PIC adopts a closed conformation which signals the termination of the scanning and the beginning of protein translation. Mutants of eIF1 in yeast that interact poorly with the PIC initiate protein synthesis at near-cognate codons and at codons in unfavorable consensus sequences [17]. eIF1 mutants increase the complexity of protein isoforms that arise from the same mRNA, emphasizing the importance of eIF1-PIC interaction to distinguishing alternative translation starts. eIF3 plays a pervasive role in initiation events. It recruits PIC to the 5′ end of the mRNA because of its affinity for eIF4G and the complex, and increased expression of eIF3 increases global translation including transcripts that are not abundantly translated in homeostasis. In yeast, mutations in eIF3 disrupt initiation as PIC fails to adopt a closed conformation on recognition of the start codon.

## eIF4E and Phosphorylation by MNK Kinases

The 5′ end cap structure is directly recognized by eIF4E, the rate-limiting component in formation of the eIF4F complex. Unexpectedly, an increase in eIF4E does not increase the global translation rate, but it alters the cellular proteome by preferentially upregulating translation of transcripts with long and structured 5′ UTR—many of which encode growth-promoting or malignancy-associated proteins [18, 19]. eIF4E is considered a central player in carcinogenesis as high levels of the protein induce oncogenic transformation in mouse fibroblasts [20], while antisense-mediated decrease in eIF4E reverses the aggressive proliferative phenotype of Ras-transformed fibrosarcomas [21]. The function of eIF4E is primarily regulated by its interaction with 4E binding protein 1 (4EBP1) as sequestration by 4EBP1 limits eIF4E availability for cap recognition and formation of the eIF4F complex. The activity of eIF4E is also in part regulated through phosphorylation at serine 209 by activated mitogen-activated protein kinase-interacting kinases (MAPK-interacting kinases), MNK1 and MNK2. The MNK enzymes are activated by RAS signaling through the downstream effector, extracellular regulated kinase (ERK) and p38 MAP kinase. MNK1/2 knockout mice, however, develop normally [22], and a lack of a phenotype in eIF4E phospho-mutant (S209A) transgenic animals suggests that the modification does not play a significant role in growth and development [23]. Unlike wild-type 4E, the expression of the phospho-mutant (S209A) in MEF increases resistance to cellular transformation, and non-phosphorylatable eIF4E mutant mice (*eIF4E*^*S*209*A*^) and MNK1/2-deficient mice are resistant to tumor progression despite a genetic background that would otherwise promote invasive cancers [23, 24]. Phosphorylated eIF4E preferentially upregulates translation of a specific set of transcripts involved in epithelial-mesenchymal transition (EMT) and metastasis, which supports the observation of increased tumor invasiveness and distant metastasis in mutant mice [25]. Mechanistic studies in a reticulocyte lysate cell-free system indicate that MNK augments eIF4E-eIF4G interaction and the assembly of eIF4F, and that phosphorylation of eIF4E preferentially facilitates the translation of mRNAs with cap and stem-loop structure at the 5′ end [26]. The mechanism by which the modification promotes translation of select mRNA is debated, but phosphorylation-dependent eIF4E sumoylation and the increase in eIF4F stability [27] is suggested to contribute to the relative increase in translation of mRNAs with structural complexity in the 5′UTR.

## Translation Regulation by mTOR-4EBP1 Signaling

The other major signaling pathway that regulates initiation is the mammalian target of rapamycin (mTOR)-4EBP1 pathway. Nutrients, insulin, and growth factors activate the pathway and signals relayed through the mTOR complex 1 (mTORC1) phosphorylate the downstream effectors ribosomal protein S6 kinase and 4EBP1. Phosphorylation of 4EBP1 reduces its binding affinity to eIF4E and increases eIF4E availability for eIF4F formation. Though activation of the MNK-eIF4E pathway and the mTOR-4EBP1 pathway promotes formation of eIF4F complex, the differential effects of MNK inhibition and mTOR inhibition suggest that eIF4F complexes assembled in each pathway target different transcript types [28]. It is also conceivable that phospho-eIF4E engages specific eIF4G and/or eIF4A isoforms on structurally-defined 5′ UTR and thus exhibits a predilection for certain transcripts. It is not surprising, then, that cell death initiated through inhibition of either pathway is less effective than inhibition of both.

## Deregulation of Translation Initiation in Cancer

**Figure 2:**
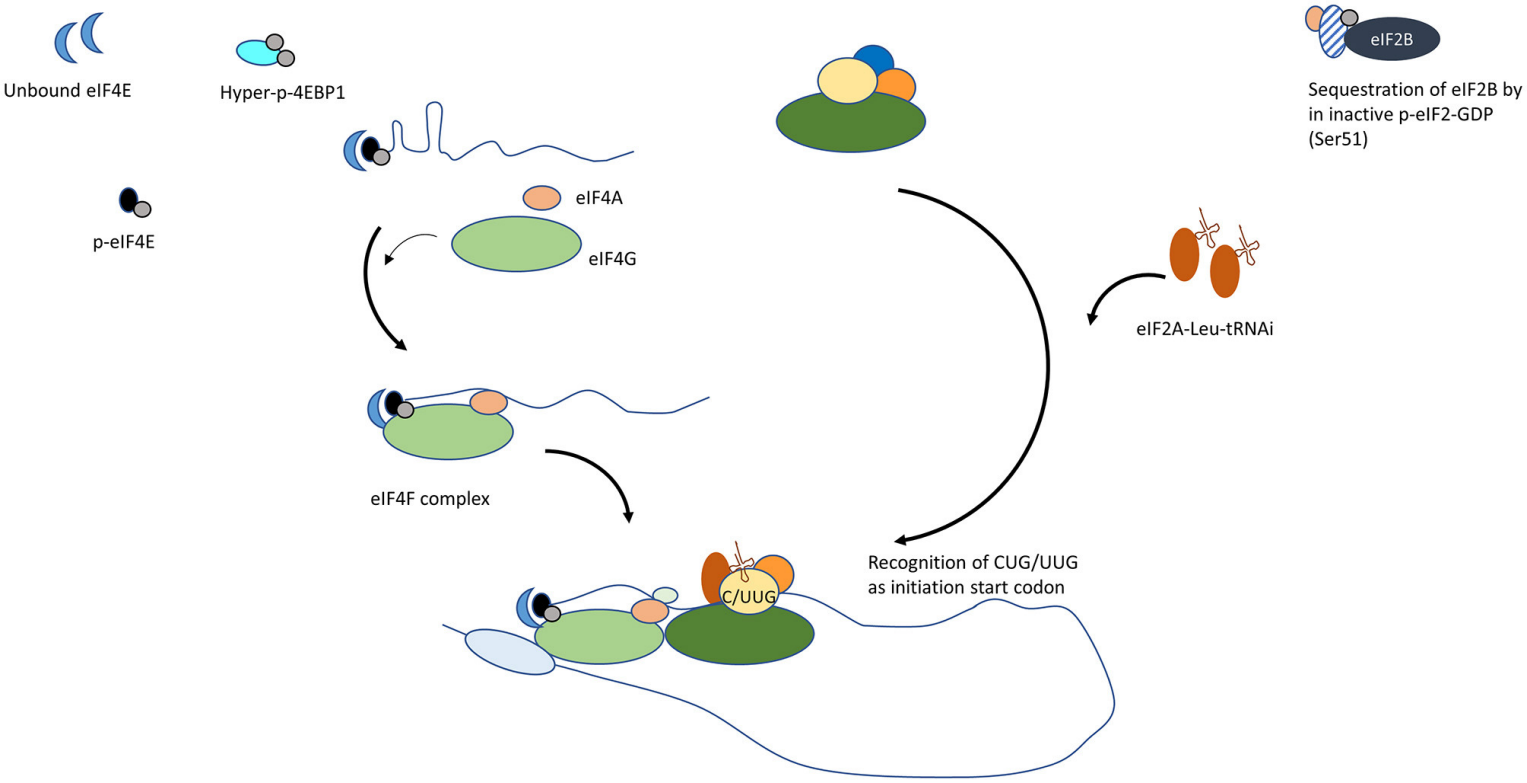
Mechanisms by which conventional translation initiation is disrupted in cancer cells. (1) Increase in unbound eIF4E. Upregulation in eIF4E transcription, reduced 4EBP or hyperphosphorylated 4EBP supports the formation of the eIF4F complex at 5′ mRNA. (2) MNK- dependent phosphorylation of eIF4E. Phospho-eIF4E promotes eIF4G interaction. (3) Increase in alternate Leu-tRNAi carrier, eIF2A. Refractory to kinases that inactivate eIF2, eIF2A engages to form PIC and initiates translation at alternative ORFs.

Initiation is a rate-limiting step in translation of capped mRNA as eIF4E is the least abundant of translation initiation factors. The altered levels of molecular factors and/or signaling pathways in cancer cells increase synthesis of non-conventional protein isoforms, resultant from aberrant translation initiation (Figure 2). Through altered abundance, cellular location, or activity of regulators of cell-cycle, apoptosis, DNA damage response and repair, and lineage fidelity, the proteome disproportionately favors reprogramming and survival of cancer cell despite a non-conducive environment [29, 30].

**Figure 3:**
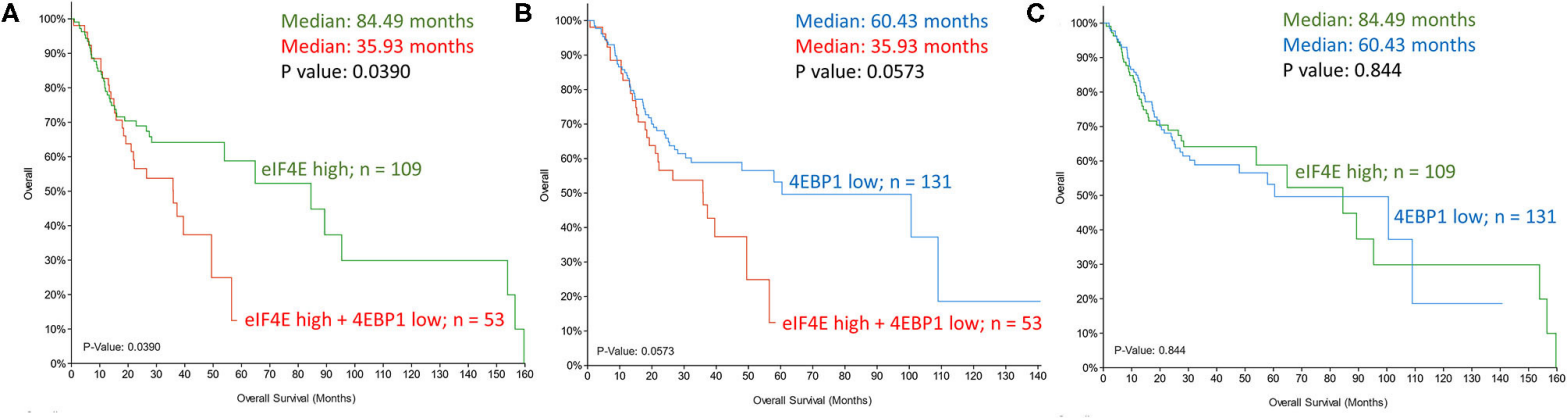
Kaplan-Meier assessment of overall survival of head and neck cancer patients from TCGA dataset. Patients were divided into cohorts based on mRNA expression z-scores relative to all samples (log RNA Seq V2 RSEM) of **(A,C)**. eIF4E (*top tercile*; *n* = 109; *green*) and **(B,C)**. 4EBP1 (*bottom tercile*; *n* = 131; *blue*) were assessed and compared to patients that satisfied both criteria (increased eIF4E and decreased 4EBP1; *n* = 53; *red*). *P*-values are shown.

A majority of head and neck squamous cell carcinoma (HNSCC) are driven by an overactive AKT-mTOR signaling pathway due to increased prevalence of PI3K mutations and PTEN loss [31, 32]. The increase in phosphorylated 4EBP1 downstream of the PIK3-AKT-mTOR signaling is an important modification that supports eIF4F formation, and inhibitors of mTOR such as rapamycin and its synthetic analogs that increase dephosphorylated levels of 4EBP1 showed promise in the treatment of HNSCC as short-term monotherapy prior to definitive treatment [33]. In a meta-analysis of 11 clinical trials, mTOR inhibitors, as single-agent therapy, failed to show a significant tumor response, and a better partial tumor response in combination with chemotherapy and/or radiotherapy needs additional evaluation to validate the sensitizing effect [34]. Rather than biallelic mutations, haploinsufficiency accounts for reduced 4EBP1 in a number of head and neck tumors [35], and TCGA database analysis demonstrates a correlation between reduced 4EBP1 expression and adverse survival outcome. Supporting this finding is the demonstration that 4EBP1/2 knockout mice are conducive to tumor growth, whereas the mutant mice with 4EBP1 that is non-phosphorylatable by mTOR limits tumor progression [35]. The central initiation factor in cap-dependent translation, eIF4E, is oncogenic when overexpressed, and multiple lines of evidence support its role in tumor formation *in vivo*. Correspondingly, high eIF4E in HNSCC tumors and adjacent margins carry poor prognostic implications [36, 37]. However, instead of a singular increase in eIF4E or a decrease in 4EBP1, there is increasing consensus that ratio eIF4E to 4EBP1 is an improved indicator of patient survival [38, 39]. mRNA expression analyses (TCGA database) support the higher predictive value of the dual mRNA signature in HNSCC (high eIF4E and low 4EBP1) relative to each independently (Figure 3). An increase in phosphorylated 4EBP1 and/or eIF4E is linked to poor prognosis in a variety of cancers including head and neck cancer [40]. MNK1/2 antagonists investigated before were potent suppressors of metastasis, but adverse effects limited their clinical transition. MNK1/2 inhibitor Tomivosertib exhibits an acceptable safety profile and, in phase II investigation, it extended progression-free survival in checkpoint inhibitor-refractory non-small cell lung cancer [41]. The inhibitor is currently in clinical evaluation for a number of advanced solid malignancies, including HNSCC (NCT03616834).

**Figure 4:**
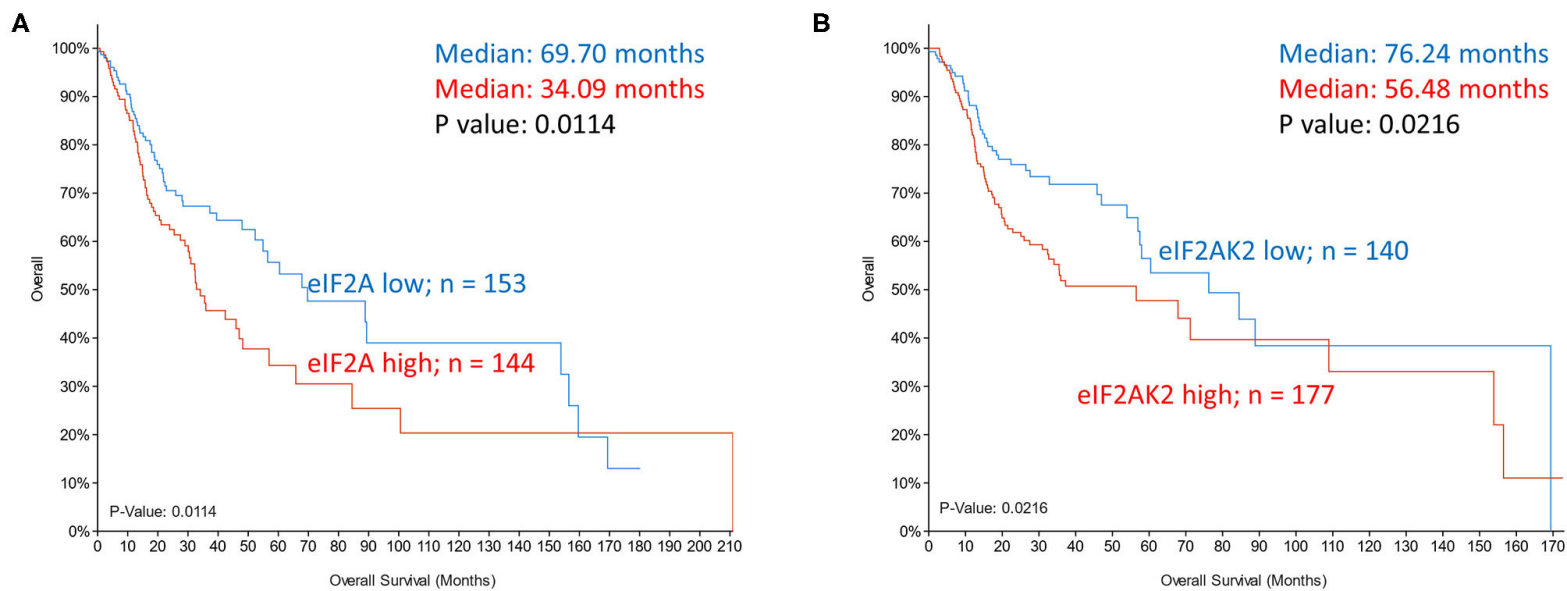
Kaplan-Meier overall survival analysis in TCGA dataset of head and neck cancer patients grouped based on mRNA expression z scores relative to all samples (log RNA Seq V2 RSEM). **(A)** eIF2A expression in *top tercile* (144 samples; *red*) and *bottom tercile* (153 samples; *blue*). **(B)** eIF2AK2 (PKR) expression in *top tercile* (177 samples; red) and *bottom tercile* (140 samples; blue). *P*-values are shown.

Global translation initiation is suppressed when eIF2α is impaired by phosphorylation. Increased phosphorylation of eIF2α Ser 51 inhibits the release of GDP-GTP exchange factor eIF2B and, in turn, the formation of the translation complex. However, increased phospho-eIF2 and limited ternary complex availability promotes leaky scanning and skipping of upstream AUG. Reassembly with the 40S complex downstream when the ternary complex is available promotes initiation instead at the downstream ORF. Although eIF2A is a poor eIF2 competitor, the alternate carrier of initiator tRNA adopts a more prominent role in cancer cells as it is refractory to eIF2α inactivating kinases [9]. eIF2A interaction with Leu-tRNAi alters the cellular proteome as initiation occurs at unconventional ORFs. The use of non-canonical start sites in transcripts that promote growth and dedifferentiation is accepted to underlie tumorigenesis [42]. FGF2 mRNA have multiple upstream ORFs and initiation at AUG and non-AUG codons generate a variety of protein isoforms that are pro-angiogenic and pro-tumorigenic [43, 44]. Non-canonical initiation codons are also responsible for different isoforms of oncogenic MYC in stress and in eIF4E transformed cells [45, 46]. It is of interest, then, that increased expression of PKR (eIF2AK2), a kinase that phosphorylates eIF2α, and of eIF2A, the recruiter of alternate tRNAi, occur in a majority of head and neck cancers (TCGA dataset) and that they significantly correlate with poor survival of HNSCC patients (Figure 4).

Stress response to viral infection inhibits protein synthesis through interferon-induced eIF2 inactivating kinase PKR. Viruses override translation inhibition to establish a conducive environment for viral replication and pathogenesis. Translational recovery in HPV infection occurs through E6 oncoprotein that facilitates dephosphorylation of eIF2α by GADD34-PP1 [47]. Reversal of eIF2 phosphorylation and of cellular rewiring of translation is suggested as a reason for improved treatment outcomes observed in HPV+ HNSCC patients.

**Figure 5:**
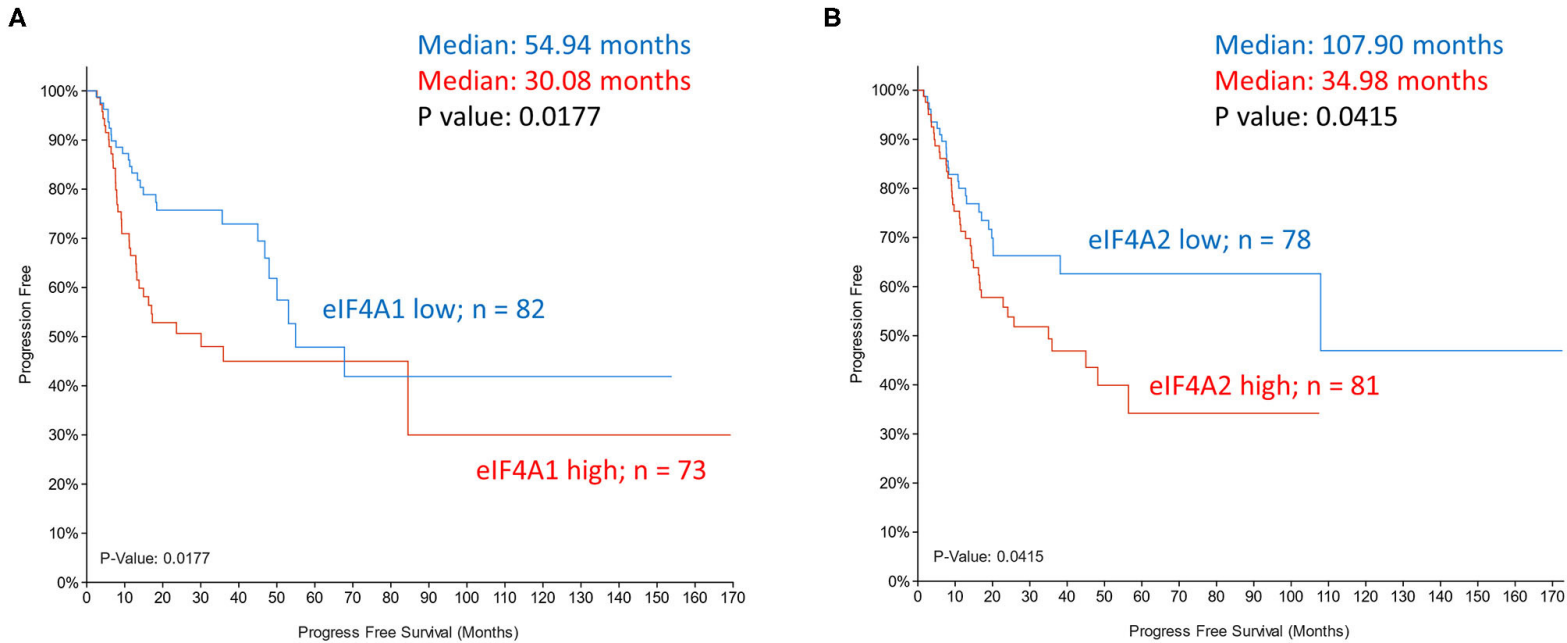
Kaplan-Meier plots of progression-free survival of head and neck cancer patients (TCGA dataset) grouped based on mRNA expression z-scores relative to all samples (log RNA Seq V2 RSEM). **(A)** eIF4A1 expression z scores >1 (samples 73; *red*) and z score <-1 (samples 82; *blue*). **(B)** eIF4A2 expression z scores >1 (samples 81; *red*) and z score <-1 (samples 78; *blue*). *P*-values are shown.

The helicase activity of eIF4A is instrumental to the unwinding of the mRNA 5′ end for docking of PIC. By stabilizing eIF4A interaction at complex mRNA structures and suppressing unwinding, inhibitors of eIF4A effectively target a subset of oncogenic mRNAs to limit tumor growth. Although eIF4A1 or eIF4A2 expression in HNSCC tumors (TCGA dataset) did not correlate with overall survival (data not shown), a significant correlation with progression-free survival was observed (Figure 5). Despite potent anti-tumor activity of eIF4A inhibitor silvestrol, pharmaceutical logistics limited its clinical evaluation. Inhibitor eFT226 (Zotatifin), suggested to have a similar mechanism of action to silvestrol, is the first eIF4A inhibitor to enter the clinical trials (NCT04092673) for advanced solid malignancies.

The dead box helicase DDX3 supports translation through recruitment of eIF3 at the 5′ end of the mRNA and it plays a critical role in unwinding complex RNA structures that impede scanning and ribosome transit. Increased expression of DDX3 promotes an aggressive phenotype in head and neck tumor cells by bypassing upstream inhibitory ORFs and initiating translation of ATF4, a transcription factor that also regulates the expression of genes associated with epithelial-mesenchymal transition [11]. In a helicase-independent manner, DDX3 drives expression of amphiregulin (AREG) and the secretory phenotype that stimulates growth of oral squamous cell carcinoma cells in an auto- paracrine mechanism [48]. Correspondingly, increased DDX3 expression, wild-type or mutant, correlates with adverse prognosis in HNSCC [11, 48]. Inhibitors of DDX3 activity are anti-tumoral in multiple cancer types; however, considering the prevalence of mutant DDX3 and helicase-independent function of DDX3, pharmacological agents that suppress DDX3 expression could have a wider clinical applicability.

Of recent, epigenetic and transcriptomic changes have gained prominence in disease prognostication, and disorder in translation control underpinning treatment challenge has garnered less attention. We briefly elaborate on a non-canonical TLK1 isoform selectively expressed in eIF4E-rich cellular milieu and its role in limiting cancer treatment efficacy.

## Tousled-Like Kinase 1

**Figure 6:**
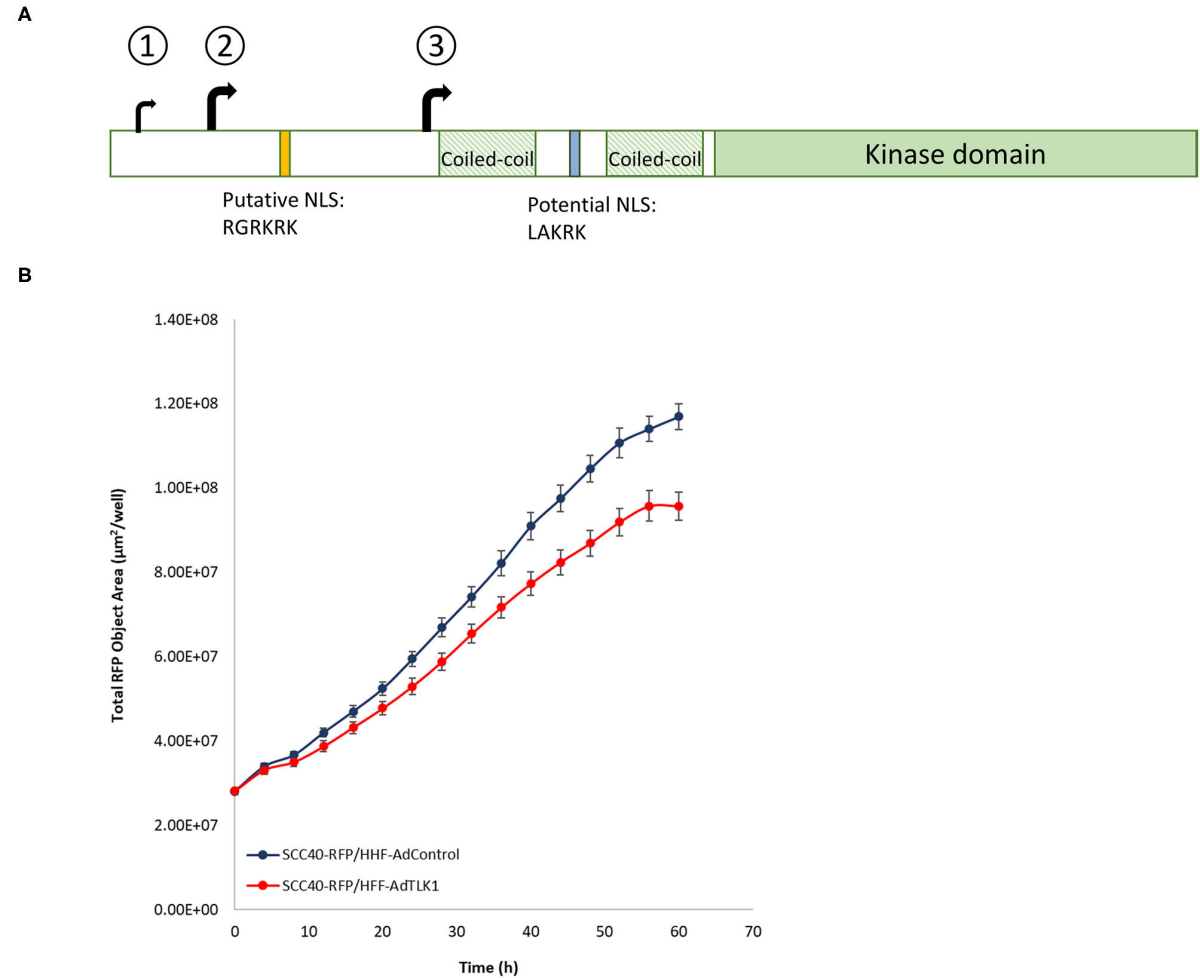
**(A)** Schematic of TLK1. In-frame translational start codons decoded by tMet-tRNAi generate isoforms that differ in the N-terminal region. Unlike AUG1, trinucleotides AUG2 and AUG3 are in near-optimal sequence context. Isoform 3 derived from translation initiation at AUG3 is devoid of putative NLS. **(B)** Lentivirus-RFP transduced SCC40 cells were puromycin-selected and RFP+ cells were sorted by FACS. Primary human foreskin fibroblasts (HFF) were transduced with control adenovirus or adenovirus-TLK1 (MOI 1000) and 24 h after transduction cells were trypsinized and resuspended with SCC40 RFP+ cells at a 1:3 ratio. Cells were co-cultured and time-lapse images acquired in the Incucyte (Sartorius) incubator. Quantification of RFP surface area from a representative experiment is shown.

TLK is evolutionarily conserved in metazoans and there are two homologs in humans: TLK1 and TLK2. TLKs are constitutively expressed in cells but their activities peak in interphase. The first identified targets of TLKs were histone chaperones ASF1A and ASF1B and expectedly, the kinases participate actively in chromatin assembly [49]. During replication halts in response to genotoxic stressors, TLK activity is transiently inhibited through phosphorylation by CHK1, and it fits the concept that kinase activity and DNA replication are intricately interconnected [50]. Not surprisingly, then, is the loss of TLK1/2 or inhibition of their activity results in unstable chromatin due to replication fork collapse and an increase in flawed transcription from heterochromatic regions of the genome [51, 52]. The presence of in-frame AUGs decoded by Met-tRNAi appear to contribute to TLK1 isoform diversity in cells. There are 3 in-frame AUGs upstream of the coiled-coiled motifs. AUG1 resides in a less-favored sequence context (TTGAUGA), and translation initiation at the following AUG, AUG2, in a near-Kozak consensus (GCAAUGG) may contribute to the full-length isoform present in most cell types (Figure 6A). Relative to the long isoform, initiation at AUG3, also in a favored context with A at −3 position (ACAAUGC), further downstream encodes a third variant that lacks a significant portion of the N-terminus region but shares identity from the two coiled-coiled dimerization motifs through to the C-terminal catalytic region. The short isoform lacks the putative nuclear localization sequence (NLS; RGRKRK) in the N-terminal region but retains a potential NLS (LAKRK) between the coiled-coiled motifs. The translation of the short isoform correlates clearly with increased levels of available eIF4E [53]. Active mTOR signaling during recovery from doxorubicin-induced DNA breaks leads to an increase in phospho-4EBP1 in murine mammary epithelial cells and the preferential translation of the shorter ORF. Abundant availability of eIF4E to drive eIF4F-driven repeat initiation, promiscuous PIC scanning due to stress-activated phosphorylation of eIF2, and limited assembly of ternary complex are factors suggested to drive TLK1 initiation at the downstream AUG3. Multiple studies in various cell types show that overexpression of the short variant augments cellular repair defenses leading to rapid recovery from genomic damage [54, 55] and, in accordance, the abundance of the isoform in breast tumors is shown to correlate with poorer patient prognosis [56, 57]. Tumor cells are reliant on the DNA repair machinery to adapt to a higher burden of genomic breaks due in part to elevated oxidative stress, a high metabolic rate, and a hypoxic environment. Mechanistically, TLK activity is critical to homology-directed repair of double-strand breaks [58] and to the stabilization of replication forks [51]. Despite the absence of the established NLS, overexpression of the shorter variant improves DNA repair kinetics, and it suggests that nuclear localization directed either by the downstream NLS or by dimerization with the full-length form contributes to the reparative phenotype. Alternatively, the variant orchestrates cellular recovery through target proteins in the cytoplasm or regulates transit of factors that promote DNA repair between the cellular compartments. Although the short TLK1 isoform phosphorylates many of the same target proteins as the full-length *in vitro* [59], the altered ratio of isoforms in cancer cells warrants investigation into the preferred substrates of isoform 3 *in vivo* that contribute to resistance to genotoxic chemotherapeutics and radiation.

Crosstalk between mesenchymal and epithelial cells during development influences cell fate and the exchange is vital to homeostasis in adulthood. Genes specifically upregulated in mammary epithelium-adjacent fibroblasts include TLKs, and conditional loss of TLK1 or TLK2 in transgenic animals induces hyperproliferation of the ductal epithelium [60]. The mechanism by which TLK1/2 regulates crosstalk, however, is a subject of ongoing investigation. Deregulated signaling from the tumor microenvironment is acknowledged to affect tumor growth, and TLK-depleted fibroblasts when co-cultured with human breast cancer cells increased cancer cell proliferation. In corollary, human fibroblasts overexpressing TLK1 limit growth of oral squamous cell carcinoma cells in co-cultures (Figure 6B). Reciprocal signaling between cancer-associated fibroblasts and cancer cells also control tumor behavior, and selectivity in smart therapeutics that suppress TLK1 in cancer cells while augmenting its expression in normal cells can improve cancer treatment response as well as limit normal tissue toxicity [54, 61].

A plethora of factors participate in translation initiation and multiple regulatory nodes in the nexus tightly control the process. Deregulation of initiation is a hallmark of cancer and translation of otherwise repressed ORFs promotes oncogenesis and contributes to aggressive, treatment-recalcitrant tumor phenotypes. An overactive AKT-mTOR pathway, reduced expression of 4EBP1, upregulated eIF2 inhibitory kinases, and increased expression of eIF2A in HNSCC patients correlate with poor patient prognosis and it suggests that redirection to non-canonical initiation renders tumors refractory to treatment. mTOR inhibitors have been extensively evaluated in HNSCC and despite an improved early outcome, the development of drug-recalcitrant tumor cells leads to recurrence [34, 62]. Tumor heterogeneity within the same tumor mass contributes to treatment refractoriness and tumor recurrence. Other than genetic and epigenetic changes, the tumor microenvironment plays an important role in cancer recurrence.

Tumor niches exposed to hypoxia, nutrient deficiency, oxidative stress, and/or shifts in pH reprogram cellular processes to improve cell fitness. Stress-induced deregulation of initiation promotes oncogenic progression. Hypoxic stress induces breast cancer aggressiveness through alternative initiation of pluripotency factors, SNAIL1, NANOG, NODAL, and like hypoxia, mTOR inhibition-induces phosphorylation of eIF2α to drive the synthesis of de-differentiation factors [63]. It is therefore appealing to speculate that an increase in cellular plasticity could underlie limited response to mTOR inhibitors in HNSCC, and it underscores the need to therapeutically target multiple deregulated translation hubs that include phospho-eIF2 to curtail the development of intrinsically resistant tumor cells.

## Author Contributions

GS-D conceived the idea, conducted the analysis, and wrote the paper.

## Funding

GS-D was supported by funds from the Feist Weiller Cancer Center. The open access publication fees was supported by the LSUHS Biochemistry Department.

## Conflict of Interest

The author declares that the research was conducted in the absence of any commercial or financial relationships that could be construed as a potential conflict of interest.

## Publisher's Note

All claims expressed in this article are solely those of the authors and do not necessarily represent those of their affiliated organizations, or those of the publisher, the editors and the reviewers. Any product that may be evaluated in this article, or claim that may be made by its manufacturer, is not guaranteed or endorsed by the publisher.
